# Detection of Foodborne Pathogens in Acute Gastroenteritis Patient’s Stool Samples Using the BioFire^®^ FilmArray^®^ Gastrointestinal PCR Panel in the Republic of Trinidad and Tobago, West Indies

**DOI:** 10.3390/microorganisms10081601

**Published:** 2022-08-09

**Authors:** Carelene Lakhan, Neela Badrie, Adash Ramsubhag, Lisa Indar

**Affiliations:** 1 Department of Food Production, University of the West Indies, St. Augustine, Trinidad and Tobago; 2 Department of Life Sciences, University of the West Indies, St. Augustine, Trinidad and Tobago; 3 The Caribbean Public Health Agency, Port of Spain, Trinidad and Tobago

**Keywords:** diarrhea, molecular diagnostic tool, enhanced foodborne pathogen testing, public health, Trinidad and Tobago

## Abstract

In 2009, the burden of illness study for acute gastroenteritis in Trinidad and Tobago highlighted that ~10% of stool samples tested were positive for a foodborne pathogen. The study also noted that limited laboratory screening for pathogens contributed to a lack of etiology as public health hospitals only routinely tested for *Salmonella* and *Shigella*, and sometimes for *Escherichia coli* and *Campylobacter*. To better understand the foodborne pathogens responsible for acute gastroenteritis, enhanced testing using the BioFire^®^ FilmArray^®^ Gastrointestinal PCR panel was used to screen diarrheal stool samples for 22 pathogens from patients in 2018. The five general public health hospitals (San Fernando, Mt. Hope, Port of Spain, Sangre Grande, and Tobago) were notified of research activities and diarrheal stool samples were collected from all acute gastroenteritis patients. A total of 66 stools were screened and ~30% of samples tested positive for a foodborne pathogen. The current study showed that a much wider range of enteric pathogens were associated with acute gastroenteritis in Trinidad and Tobago than previously reported in 2009. These findings can be used by health officials to guide appropriate interventions, as well as to provide evidence for adoption of the PCR panel detection method at public health hospitals to benefit patient care.

## 1. Introduction

Acute gastroenteritis is an inflammation of the gastrointestinal tract that may result in the sudden onset of symptoms such as abdominal pain, cramping, nausea, vomiting, and diarrhea. This illness, ranging from mild to severe, is usually caused by consumption of food contaminated with harmful bacteria, viruses, parasites, or chemical substances and is responsible for significant cases of morbidity, disability, and mortality globally [1,2]. The World Health Organization (WHO) estimated that approximately one in ten persons globally fall ill and more than 6 billion global cases of diarrheal illness occur annually [3]. The illness was also reported to affect nearly 135,000 residents in Trinidad and Tobago each year and is one of the most flagged syndromes for the Caribbean, causing significant health and economic burdens in the region [4,5] Globally, the most common reported foodborne pathogens transmitted to humans through contaminated food were *Salmonella, Campylobacter, Shigella,* pathogenic *Staphylococcus aureus, Escherichia coli* 0157:H7, *Yersinia, Bacillus, Listeria monocytogenes*, *Vibrio*, rotavirus, and norovirus [6,7,8]. Surveillance data from the Caribbean have reported an increase in the number of acute gastroenteritis cases, foodborne disease outbreaks, and related pathogens over the last two decades, with non- typhoidal *Salmonella, Ciguatera* poisoning, *Salmonella typhi*, *Shigella*, *Campylobacter*, norovirus, and pathogenic *Escherichia coli* causing most infections [6]. The capacity to accurately identify these causative foodborne pathogens and others is critical to finding or prescribing effective patient treatment, as well as to assist in determining etiology during foodborne outbreaks for quick control and prevention of future occurrences [9].

In 2009, the burden of illness for acute gastroenteritis was estimated in Trinidad and Tobago. In that study, the five general public health hospitals (San Fernando, Mt. Hope, Port of Spain, Sangre Grande, and Tobago) which provide health care to the majority of the population, processed and tested >80% of all acute gastroenteritis ill patient’s stool samples; laboratory findings indicated that 10% of all samples tested positive for a foodborne pathogen, and the main method of screening stools was by use of traditional culture practices which provided limited data on the etiology of illness as they only “routinely” tested for *Salmonella* and *Shigella* and “sometimes” tested for *E. coli* and *Campylobacter* based on the physician’s request [5]. The use of molecular techniques and other nucleic acid-based methods, even though more costly than traditional culture techniques, were reported to offer highly sensitive and specific automated reliable results with shorter turnaround times [10]. Further analyses involving the use multiplex molecular assays for the detection of common foodborne pathogens in stool samples observed that these panels detected the most gastrointestinal pathogens when compared to conventional methods, and that the Food and Drug Association-approved PCR-based test panel, the BioFire^®^ FilmArray^®^ Gastrointestinal (GI) Panel (BioFire Diagnostics, Salt Lake City, UT, USA), was a top performer among other molecular-based detection tools [10,11,12]. The BioFire FilmArray test panel was also reported to rapidly detect and identify foodborne pathogen genes in ill patients, which resulted in shorter turn-around times, with accurate, highly sensitive (98.5%), and specific (99.2%) results to benefit patient care [13,14,15].

A review of current research indicated that there were no follow-up studies conducted after 2009 with regards to enhanced testing for a wider range of foodborne pathogens from acute gastroenteritis patients in Trinidad and Tobago. The ability to test for such pathogens is critical to expand on the etiology of this illness since the current testing method only provides limited data for approximately four (4) pathogens. Use of the BioFire FilmArray PCR panel in this study can be useful to provide the critical etiological data being sought as this PCR panel can test for thirteen (13) foodborne bacteria, five (5) viruses, and four (4) parasites per acute gastroenteritis patient’s stool sample. Additionally, literature searches on the use of this PCR panel in Trinidad and Tobago or even the wider Caribbean Region could not be found, and therefore, this research could be considered novel. Hence, the data obtained in this study could be used to assist in guiding health officials to prevent and treat this illness, as well as to provide baseline data for future investigative studies on acute gastroenteritis in Trinidad and Tobago. The objectives of this study were, therefore, to determine by detection the pathogens responsible for acute gastroenteritis through enhanced testing using the PCR panel, and to even further recommend whether its use could be adopted for future testing at public health hospitals in Trinidad and Tobago.

## 2. Materials and Methods

### 2.1. Research Design

Diarrheal stool samples were prospectively collected from patients (<5 years old and >5 years old) diagnosed with acute gastroenteritis at the five general public hospitals in Trinidad and Tobago and screened for 22 foodborne pathogens in 2018 using the BioFire FilmArray PCR panel.

### 2.2. Assumption

This study assumed that the doctor’s diagnosis was accurate when patients presented at public health hospitals for acute gastroenteritis based on the case definition of “>3 loose stools in a 24 h period and other typical symptoms of acute gastroenteritis”. Therefore, all stool samples submitted and tested were due to this illness.

### 2.3. Ethical Approval

Ethical approval for this study was granted from the Ethical Review Board of the University of the West Indies and the Review Committee of the Ministry of Health, Trinidad and Tobago. Each participant was informed of the purpose of the study and all data collected were kept confidential.

### 2.4. Collection of Stool Samples for Pathogen Testing and Sample Size

In January 2018, doctors and nurses at the five general public health hospitals (San Fernando, Mt. Hope, Port of Spain, Sangre Grande, and Tobago) were requested to collect a stool sample from all patients (<5 years old and >5 years old) diagnosed with acute gastroenteritis at their facility for a one-year prospective study. These major hospitals provided a good representative cross-section of the 1,300,000 residents in Trinidad and Tobago who opt to seek healthcare for their illnesses. The case definition for an acute gastroenteritis patient was “the sudden onset of diarrhea, with or without fever (>38 °C or 100.4 °F) and presenting with 3 or more loose/watery stools in the past 24 h, with or without dehydration, vomiting, and/or visible blood”. Therefore, in 2018 the sample size included all patients presenting at the public health institution with 3 or more loose/watery stools in a 24 h period. Once the patient agreed to partake in the study, their demographic data along with a fresh stool sample (approximately 1–25 g of liquid stools and/or rectal swabs) were collected and immediately screened for foodborne pathogens according to established laboratory protocols and the manufacturer’s recommended methodology for the PCR panel [16]. During the months of January to December 2018, sample collection was sparse, averaging 5–6 samples per month as many persons tend to self-treat for acute gastroenteritis and even with several sensitization sessions held with the public health hospitals’ personnel, patients seeking healthcare were still reluctant to submit stool samples, and for those patients who opted to seek private healthcare, they would not have been captured by the public hospitals [17]. A total of 66 diarrheal stools were prospectively collected from patients (<5 years old and >5 years old) diagnosed with acute gastroenteritis at the five general public hospitals and screened for 22 foodborne pathogens in 2018.

### 2.5. Polymerase Chain Reaction Panel

#### 2.5.1. Foodborne Pathogens for Testing

The pathogens for testing with the BioFire Gastrointestinal Panel were preselected and included: *Campylobacter* (*jejuni, coli,* and *upsaliensis*); *Clostridium difficile* (toxin A/B); *Plesiomonas shigelloides*; *Salmonella*; *Yersinia enterocolitica*; *Vibrio* (*parahaemolyticus, vulnificus*, and *cholerae*); *Vibrio cholerae*; Enteroaggregative *E. coli* (EAEC); Enteropathogenic *E. coli* (EPEC); Enterotoxigenic *E. coli* (ETEC) lt/st; Shiga-like toxin-producing *E. coli* (STEC) stx1/stx2; *E. coli* O157; Shigella/Enteroinvasive *E. coli* (EIEC); adenovirus F40/41; astrovirus; norovirus GI/GII; rotavirus A; sapovirus (I, II, IV, and V); *Cryptosporidium*; *Cyclospora cayetanensis*; *Entamoeba histolytica;* and *Giardia lamblia*.

#### 2.5.2. Detection of Foodborne Pathogens

The PCR panel is an automated in-vitro diagnostic (IVD) system which uses nested multiplex PCR and high-resolution melting analysis to detect and identify multiple nucleic acid targets from the diarrheal stool samples. Nest multiplex PCR utilizes two stages of PCR. In the first stage, multiple outer primers perform multiplex PCR on the target template that are present in the sample, while the second stage PCR is performed in a singleplex manner, further amplifying the DNA procured during the first stage PCR. The inner primers which are used in the second-stage PCR are made of sequences “nested” within the first-stage PCR products. LC Green^®^ Plus is used as the binding dye which is incorporated into copies of DNA as they are produced during each PCR cycle. After the last stage PCR, the instrument gradually increases the temperature of the reaction to 72 °C and the copies of the double stranded DNA melt. The LC Green^®^ Plus dye is then released and a decrease in the fluorescence is detected by the FilmArray instrument. The time taken to complete the test is <2 h [16]. 

### 2.6. Data Analysis

Descriptive statistics to summarize the characteristics of the dataset in terms of foodborne pathogens detected from acute gastroenteritis patients who sought health care were performed using SPSS 24 (IBM Corp., Armonk, NY, USA).

## 3. Results and Discussion

### 3.1. Foodborne Pathogens Detected Using the PCR Panel from Stool Samples

**Figure 1:**
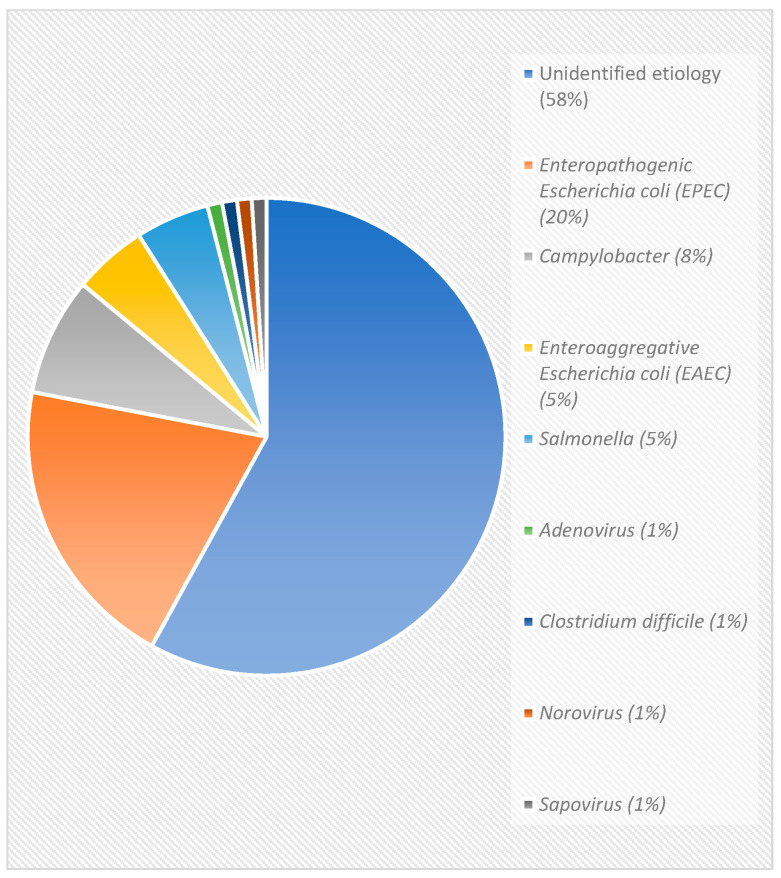
Foodborne pathogens detected using PCR in Trinidad and Tobago, 2018.

In 2018, based on a doctor’s accurate diagnosis for acute gastroenteritis, all stool samples from all patients (*n* = 66) diagnosed with the illness were each tested for 22 pathogens, and nearly 30% (*n* = 20) of patients tested positive for a foodborne pathogen. The major pathogens detected were *Enteropathogenic E. coli* (~20%), *Campylobacter* (~8%)*, Salmonella* (~5%), and *Enteroaggregative E. coli* (~5%), as seen in Figure 1, indicating that there are other pathogens detected to cause acute gastroenteritis illness apart from the ones routinely tested for at the public hospitals. This was the first recorded study in Trinidad and Tobago, and the wider Caribbean Region, on the use of the BioFire Gastrointestinal Panel to test for foodborne pathogens from stool samples submitted by patients ill with acute gastroenteritis.

### 3.2. Foodborne Pathogens Responsible for Acute Gastroenteritis Illness in Trinidad and Tobago

Data generated in this research differed in both sample size and detection method from the 2009 study, and while direct comparisons could not be drawn, the pathogens detected from both studies suggested that there are more pathogens responsible for acute gastroenteritis in Trinidad and Tobago than previously reported and currently tested. According to data sourced from the Caribbean Public Health Agency’s Laboratory Information System (LABIS), foodborne pathogens reported by the Ministry of Health, Trinidad and Tobago, for the period 2006–2016 included adenovirus, *Campylobacter, E. coli*, norovirus, *Salmonella*, and *Shigella.* Thus, even with a small sample size in 2018, the additional etiological information gathered from this research can still provide a baseline for further studies to assist health officials in guiding appropriate interventions.

Foodborne bacteria were the most prevalent pathogens detected in stool samples from ill patients in this study and were also reported to be responsible for 10% to 55% of all diarrheal cases in developing countries that caused more severe cases of acute gastroenteritis [18,19]. In 2009, *Salmonella* was the leading pathogen responsible for acute gastroenteritis in Trinidad and Tobago, while in 2018, many patients were co-infected with two bacteria spp., namely *EPEC* and *Campylobacter.* EPEC and other mono infections are usually self-limiting but co-infections with other pathogens were reported to increase the severity of this illness [12,20,21,22]. 

Identification of foodborne pathogens is crucial to controlling and preventing related illnesses and associated burdens [23]. Data reported in this research and by LABIS indicated that in Trinidad and Tobago, the foodborne pathogens detected to cause acute gastroenteritis were *Salmonella, Shigella*, norovirus, rotavirus, *Campylobacter, Clostridium difficile, E. coli*, adenovirus, and sapovirus. The Centre for Disease Control and Prevention (CDC) reported that, in the USA, adult bacterial related infections were often associated with travel and pathogens such as *Salmonella*, *Campylobacter*, *Shigella*, and Shiga toxin-producing *Escherichia coli* (enterohemorrhagic *E. coli)* were identified as the cause [24]. Norovirus was identified as the leading cause of viral gastroenteritis in the adult population, while in children most cases (70%) were caused by rotavirus and norovirus; ~10% by protozoa; and ~10% to 20% by bacteria [25,26,27]. One diarrheal disease study in children also reported that even though bacterial pathogens were the major group of organisms responsible for the disease, rotavirus was still the single largest causative agent [28]. Generally, many foodborne pathogens can be transmitted in the same way, including animal to human interaction, consuming contaminated food or drink, ill food handlers, and transfer by contaminated surfaces and utensils [29,30]. In addition, with increased international travel and globalization of the food industry, all foodborne pathogens should be monitored and appropriate preventative strategies such as adequate sanitation, food hygiene, targeted health education, and access to safe water for drinking should be encouraged [31,32]. 

Our research also found that most (58%) stool samples tested did not have a definitive causal agent. This finding was consistent with reports by the CDC in that unidentified etiology was reported to contribute to more episodes of acute gastroenteritis and foodborne illnesses than detected pathogens [33]. Possible reasons for unidentified causal agents in Trinidad and Tobago may include limited pathogen screening as almost 31 foodborne enteric pathogens were identified and reported to cause illness and our study screened for 22 [34]. Hence, pathogens such as *Bacillus cereus, Brucella* spp., *Listeria monocytogenes, Mycobacterium bovis, Staphylococcus aureus, Streptococcus* spp. group A, *Toxoplasma gondii*, *Trichinella* spp., and hepatitis A virus, which were not tested for in our study, could have been other causative agents. Other possibilities for not identifying a causal agent include that: it may not be a pathogen in the first place but perhaps a toxin or some underlying condition of the patient; the casual organism was no longer present within the patient’s system since diarrhea is the body’s mechanism to expel toxic materials; or the agent responsible has not yet been identified or remains unproven as causing a foodborne illness [35,36,37,38]. Therefore, with continuous surveillance, research activities, and testing for a wider range of pathogens, the gap between identified and unidentified could be narrowed.

### 3.3. Use of the PCR Panel in Public Health Hospitals, Trinidad and Tobago

The detection rate for foodborne pathogens in our study was lower than that reported in other studies, but so was our sample size [11,39,40,41,42]. Nevertheless, this PCR panel detected a positive pathogen in ~30% of all samples tested in less than 2 h. The detection of sapovirus in this study was also noteworthy as limited to no data currently exist for this pathogen as an important cause of acute gastroenteritis in Trinidad and Tobago and considering the growing awareness for sapoviruses in child healthcare, further investigative studies are needed [43]. The traditional culture testing method presently employed at the public health hospitals generally yields results after 24 h and was reported in past studies to be erroneous, time consuming, and at the discretion of physicians’ request [44,45,46]. Use of this PCR panel was also described to rapidly and accurately identify causal pathogens and coinfections, which resulted in the administration of treatments sooner; reduced antibiotic use for “just in case” scenarios; targeted antibiotics therapy started sooner; shorter hospital stays; and reduced morbidity/mortality associated with foodborne illnesses [13,15,47]. Further investigations regarding turnaround times for collection of stools from patients and initiating therapy with cost analysis are also needed, but until then, this PCR panel can be introduced and used at all public health hospitals in Trinidad and Tobago to benefit patient care.

### 3.4. Demographic Factors and Foodborne Pathogens Detected from Acute Gastroenteritis Patients in Trinidad and Tobago

In 2018, more samples were submitted from acute gastroenteritis patients who were >5 years old, males, and attended the Sangre Grande General Hospital (Table 1). Eight of the positive patients were co-infected with two pathogens, and most co-infections were observed from >5-year-old males who sought health care at the Sangre Grande General Hospital (Table 2). In our study, 100% of co-infections observed involved EPEC and another bacterium. This finding was higher than other studies involving two or more organisms detected which involved EPEC and EAEC [15,41,48]. Similar to our study, one study involving ten participating clinical microbiology laboratories in Austria, Finland, France, Germany, Greece, Ireland, Italy, Portugal, Romania, and the UK, and other studies in Costa Rica and South India, also commonly observed high co-infection rates of *Campylobacter* with EPEC [49,50,51]. However, such studies failed to provide explanations for these findings or whether microbes were more likely or less likely to occur together or if cases were population specific [20,52,53,54,55]. Thus, high coinfection rates indicated varied but common etiology for acute gastroenteritis in Trinidad and Tobago, which warrants further investigations using larger datasets to explain these findings. All *Campylobacter* and ~62% of *EPEC* pathogens detected were from patients who sought health care at the Sangre Grande General Hospital (Table 2). The catchment population for the Sangre Grande General Hospital is ~120,000 residents spanning from Matelot in the North to Guayaguayare, Rio Claro, and Brothers Road in the South to Valencia in the East and covering approximately one-third of the island. This area is sparsely populated and some districts within are considered to have poverty levels higher than the national level. Two-thirds of this region are rural with agriculture (fishing and farming, including chicken farms) as major activities [56,57]. In 2018, the National Meteorological Station reported major flooding activities that affected many residents in this area [58]. All these factors may increase the risk of these residents to *Campylobacter* infections since multiple studies have reported more human *Campylobacter* infections in rural areas when compared to urban, with occupation (agricultural related work); handling of livestock (chickens, cattle); and exposure to contaminated water sources from flooding episodes as source attributions [59,60,61,62,63]. Strategies suggested by the Center of Disease Control and Prevention (CDC) to mitigate the risks of infection in a catchment area like this include targeted educational campaigns such as disease awareness and proper hygiene techniques among agricultural workers; cleaning and sanitizing of hands, tools, and equipment; and not drinking untreated water, especially after flooding episodes. However, further investigations with a larger sample size are recommended to determine whether associations between locations and pathogens occur as samples were collected during different months in 2018 and could suggest infection was not a cluster with one source.

Enteropathogenic *Escherichia coli* (EPEC) is a gram-negative bacterium that adheres to intestinal epithelial cells and causes diarrhea, with outbreaks reported mainly among children in pediatric wards and day-care centers [64,65,66,67]. Even though typical strains of EPEC were reported to cause infantile diarrhea within the community and hospital settings, in our study nearly 70% of patients were over the age of 5 years, which may suggest new possible strains of this pathogen-with both humans and animals as reservoirs, and therefore, further investigations with larger sample sizes are needed to confirm if this is the situation in Trinidad and Tobago [68,69,70,71]. Additionally, EPEC strains in Trinidad and Tobago should be further characterized to determine whether new pathotype or atypical EPEC strains exist. Targeted efforts to reduce infection rates by this pathogen in Trinidad and Tobago include proper hygiene, especially when interacting with animals.

## 4. Conclusions

Acute gastroenteritis can affect any person at any time along the farm to table continuum but may be prevented once proper food safety systems are in place. To better understand and guide appropriate interventions to prevent this illness, routine monitoring; surveillance activities; and identification of pathogens are essential. In our study, even though the sample size was small, there were other pathogens detected to cause acute gastroenteritis illness apart from the ones routinely tested for at the public hospitals. The pathogens identified in this research that caused illness included: *Salmonella, Shigella*, norovirus, rotavirus, *Campylobacter, Clostridium difficile, E. coli*, adenovirus, and sapovirus. It was observed that more samples were submitted from acute gastroenteritis patients who were >5 years old, males, and attended the Sangre Grande General Hospital. Further investigative studies are required to establish any relationship between location and pathogens detection from ill patients seeking healthcare.

All foodborne pathogens detected can be transmitted in similar ways, including by animal to human interaction, consuming contaminated food or drink, ill food handlers, and transfer by contaminated surfaces and utensils. To mitigate the risk of this illness, appropriate preventative strategies such as adequate sanitation, food hygiene, targeted health education, and access to safe water for drinking are needed. This research was the first recorded study in Trinidad and Tobago and the wider Caribbean Region on the use of the BioFire gastrointestinal panel to test for foodborne pathogens from stool samples submitted by patients ill with acute gastroenteritis. This PCR panel allowed for the fast detection of a wide range of pathogens including sapovirus, for which there are no reported studies conducted in Trinidad and Tobago examining it as a cause of acute gastroenteritis, as well as coinfections with two pathogens which would probably not have been detected given the present diagnostic method at the public health hospitals. Hence, data from this study can be used as a baseline in future acute gastroenteritis etiological studies, provide evidence for health officials to guide interventions for the prevention of future foodborne related illnesses, and recommend the adoption and use of this PCR panel at each public hospital to allow for better patient care in terms of the administration of appropriate treatments sooner and ultimately an overall decrease in morbidity/mortality associated with foodborne illnesses in Trinidad and Tobago.

## 5. Limitations

Due to ethical reasons and patient confidentiality, patient data/history were limited and as such more rigorous analyses/inferences could not be made.

## Figures and Tables

**Table 1:** Demographic factors associated with foodborne pathogens detected via PCR panel from acute gastroenteritis patients in Trinidad and Tobago, 2018.

Demographics Factors		Foodborne Pathogens Detected via PCR in Trinidad and Tobago, 2018 (*n* = 28)							
		*Campylobacter*	*Clostridium difficile*	*Salmonella*	*EAEC*	*EPEC*	*Adenovirus*	*Norovirus*	*Sapovirus*
Age group	<5	1	0	0	2	4	0	0	0
	>5	4	1	3	1	9	1	1	1
Sex	Female	1	0	2	0	5	0	0	1
	Male	4	1	1	3	8	1	1	0
Public Health Hospital	Sangre Grande	5	0	1	2	8	0	0	0
	Mt. Hope	0	1	1	0	2	1	1	1
	Port of Spain	0	0	0	1	3	0	0	0
	San Fernando	0	0	1	0	0	0	0	0
	Tobago ᶲ	-	-	-	-	-	-	-	-

ᶲ No samples received from the Tobago General Hospital.

**Table 2:** Co-infections detected via PCR panel from acute gastroenteritis patients in Trinidad and Tobago, 2018.

Demographic Factors			Co-Infections Detected via PCR from Acute Gastroenteritis in Trinidad and Tobago, 2018 (*n* = 8)
Age Group	Sex	Public Health Hospitals	Foodborne Pathogens
>5 years	Male	Sangre Grande	EPEC and *Campylobacter*
<5 years	Female	Sangre Grande	EPEC and *Campylobacter*
>5 years	Male	Sangre Grande	EPEC and *Campylobacter*
>5 years	Male	Sangre Grande	EPEC and *Campylobacter*
>5 years	Male	Sangre Grande	EPEC and *Campylobacter*
>5 years	Female	Sangre Grande	EPEC and *Salmonella*
<5 years	Male	Mt. Hope	EPEC and EAEC
<5 years	Male	Sangre Grande	EPEC and EAEC

## Data Availability

The data presented in this study are available upon request from the corresponding author. The data are not publicly available due to ethical reasons involving patient confidentiality.

## Abbreviations

The following abbreviations are used in this manuscript:

PCR	Polymerase chain reaction
PCR panel	BioFire^®^ FilmArray^®^ Gastrointestinal Panel Multiplex PCR Test

## References

[1] Global Burden of Disease (GBD) 2016 Causes of Death Collaborators (2017). Global, regional, and national age-sex specific mortality for 264 causes of death, 1980-2016: A systematic analysis for the Global Burden of Disease Study 2016. Lancet.

[2] Papadopoulos T., Klamer S., Jacquinet S., Catry B., Litzroth A., Mortgat L., Mamouris P., Rebolledo J., Vaes B., Van Cauteren D. (2019). The health and economic impact of acute gastroenteritis in Belgium, 2010–2014. Epidemiol. Infect..

[3] World Health Organization (WHO) (2021). Newsroom-Fact-Sheets: Food Safety Key Facts. https://www.who.int/NEWS-ROOM/FACT-SHEETS/DETAIL/FOOD-SAFETY.

[4] Caribbean Public Health Agency (CARPHA) (2017). Monthly Syndromic Surveillance Summary. https://carpha.org/Portals/0/Documents/MSSS_No3_EpiWeek_8-11-2017.pdf.

[5] Lakhan C., Badrie N., Ramsubhag A., Sundaraneedi K., Indar L. (2013). Burden and impact of acute gastroenteritis and foodborne pathogens in Trinidad and Tobago. J. Health Popul. Nutr..

[6] Caribbean Public Health Agency (CARPHA) (2017). State of Public Health in the Caribbean Region 2014–2016: Building Resilience to Immediate and Increasing Threats: Vector-Borne Diseases and Childhood Obesity.

[7] Bhunia A.K. (2018). Foodborne Microbial Pathogens: Mechanisms and Pathogenesis.

[8] Pires S.M., Desta B.N., Mughini-Gras L., Mmbaga B.T., Fayemi O.E., Salvador E.M., Gobena T., Majowicz S.E., Hald T., Hoejskov P.S. (2021). Burden of foodborne diseases: Think global, act local. Curr. Opin. Food Sci..

[9] Centre for Disease Control and Prevention (CDC) (2022). Foodborne Outbreaks: You Can Help Solve Foodborne Outbreaks. https://www.cdc.gov/foodsafety/outbreaks/investigating-outbreaks/help-solve-outbreaks.html.

[10] Law J.W., Ab Mutalib N.S., Chan K.G., Lee L.H. (2015). Rapid methods for the detection of foodborne bacterial pathogens: Principles, applications, advantages and limitations. Front. Microbiol..

[11] Huang R.S., Johnson C.L., Pritchard L., Hepler R., Ton T.T., Dunn J.J. (2016). Performance of the Verigene® enteric pathogens test, Biofire FilmArray™ gastrointestinal panel and Luminex xTAG^®^ gastrointestinal pathogen panel for detection of common enteric pathogens. Diagn. Microbiol. Infect. Dis..

[12] Zhang S.X., Zhou Y.M., Xu W., Tian L.G., Chen J.X., Chen S.H., Dang Z.S., Gu W.P., Yin J.W., Serrano E. (2016). Impact of co-infections with enteric pathogens on children suffering from acute diarrhea in southwest China. Infect. Dis. Poverty.

[13] Foddai A., Grant I.R. (2020). Methods for detection of viable foodborne pathogens: Current state-of-art and future prospects. Appl. Microbiol. Biotechnol..

[14] Maurer F.P., Christner M., Hentschke M., Rohde H. (2017). Advances in Rapid Identification and Susceptibility Testing of Bacteria in the Clinical Microbiology Laboratory: Implications for Patient Care and Antimicrobial Stewardship Programs. Infect. Dis. Rep..

[15] Torres-Miranda D., Akselrod H., Karsner R., Secco A., Silva-Cantillo D., Siegel M.O., Roberts A.D., Simon G.L. (2020). Use of BioFire FilmArray gastrointestinal PCR panel associated with reductions in antibiotic use, time to optimal antibiotics, and length of stay. BMC Gastroenterol..

[16] FilmArray® Panels, Gastrointestinal Panel (2018). Documents: Gastrointestinal Tech Notes. https://www.biofiredx.com/products/the-filmarray-panels/#gastrointestinal.

[17] Rudzik A.E. (2003). Examining health equity through satisfaction and confidence of patients in primary healthcare in the Republic of Trinidad and Tobago. J. Health Popul. Nutr..

[18] Bintsis T. (2017). Foodborne pathogens. AIMS Microbiol..

[19] Sattar S.B.A., Singh S. (2021). Bacterial Gastroenteritis.

[20] Bhavnani D., Goldstick J.E., Cevallos W., Trueba G., Eisenberg J.N. (2012). Synergistic effects between rotavirus and coinfecting pathogens on diarrheal disease: Evidence from a community-based study in northwestern Ecuador. Am. J. Epidemiol..

[21] Valentini D., Vittucci A.C., Grandin A., Tozzi A.E., Russo C., Onori M., Menichella D., Bartuli A., Villani A. (2013). Coinfection in acute gastroenteritis predicts a more severe clinical course in children. Eur. J. Clin. Microbiol. Infect. Dis..

[22] Vergadi E., Maraki S., Dardamani E., Ladomenou F., Galanakis E. (2021). Polymicrobial Gastroenteritis in Children. Acta Paediatr..

[23] Wei X., Zhao X. (2021). Advances in typing and identification of foodborne pathogens. Curr. Opin. Food Sci..

[24] Centre for Disease Control and Prevention (CDC) (2020). Foodborne Germs and Illnesses: Causes of Food Poisoning. https://www.cdc.gov/foodsafety/foodborne-germs.html.

[25] Chow C.M., Leung A.K.C., Hon K.L. (2010). Acute gastroenteritis: From guidelines to real life. Clin. Exp. Gastroenterol..

[26] Krenzer M.E. (2012). Viral gastroenteritis in the adult population: The GI peril. Crit. Care Nurs. Clin. N. Am..

[27] O’Shea H., Blacklaws B.A., Collins P.J., McKillen J., Fitzgerald R. (2019). Viruses Associated with Foodborne Infections. Ref. Modul. Life Sci..

[28] Dadonaite, Bernadeta, Hannah Ritchie and Max Roser (2018). Diarrheal Diseases. https://ourworldindata.org/diarrheal-diseases.

[29] Oliver S.P. (2019). Foodborne Pathogens and Disease, Special Issue on the National and International PulseNet Network. Foodborne Pathog. Dis..

[30] Stein R.A., Chirilã M. (2017). Routes of Transmission in the Food Chain. Foodborne Dis..

[31] FoodSafety.gov (FSGov) (2020). Keep Food Safe: Four (4) Steps to Food Safety. https://www.foodsafety.gov/keep-food-safe/4-steps-to-foodsafety.

[32] World Health Organization (WHO) WHO Fact Sheets—Diarrhoeal Disease 2017. https://www.who.int/news-room/fact-sheets/detail/diarrhoeal-disease.

[33] Burke R.M., Mattison C.P., Marsh Z., Shioda K., Donald J., Salas S.B., Naleway A.L., Biggs C., Schmidt M.A., Hall A.J. (2021). *Norovirus* and Other Viral Causes of Medically Attended Acute Gastroenteritis Across the Age Spectrum: Results from the Medically Attended Acute Gastroenteritis Study in the United States. Clin. Infect. Dis..

[34] Adley C.C., Ryan M.P. (2016). The Nature and Extent of Foodborne Disease. Antimicrobial Food Packaging.

[35] Franklin N., Hope K., Glasgow K., Glass K. (2020). Describing the epidemiology of foodborne outbreaks in New South Wales from 2000 to 2017. Foodborne Pathog. Dis..

[36] Jahan S. (2012). Epidemiology of foodborne illness. Sci. Health Soc. Asp. Food Ind..

[37] Scallan E., Griffin P.M., Angulo F.J., Tauxe R.V., Hoekstra R.M. (2011). Foodborne Illness Acquired in the United States—Unspecified Agents. Emerg. Infect. Dis..

[38] Smith J.L., Fratamico P.M. (2018). Emerging and re-emerging foodborne pathogens. Foodborne Pathog. Dis..

[39] Murphy C.N., Fowler R.C., Iwen P.C., Fey P.D. (2016). Evaluation of the BioFire FilmArray gastrointestinal panel in a midwestern academic hospital. Eur. J. Clin. Microbiol. Infect. Dis..

[40] Park S., Hitchcock M.M., Gomez C.A., Banaei N. (2017). Is follow-up testing with the FilmArray gastrointestinal multiplex PCR panel necessary?. J. Clin. Microbiol..

[41] Piralla A., Lunghi G., Ardissino G., Girello A., Premoli M., Bava E., Arghittu M., Colombo M.R., Cognetto A., Bono P. (2017). FilmArray™ GI panel performance for the diagnosis of acute gastroenteritis or hemorragic diarrhea. BMC Microbiol..

[42] Stockmann C., Pavia A.T., Graham B., Vaughn M., Crisp R., Poritz M.A., Rogatcheva M. (2016). Detection of 23 gastrointestinal pathogens among children who present with diarrhea. J. Pediatric Infect. Dis. Soc..

[43] Becker-Dreps S., Bucardo F., Vinjé J. (2019). Sapovirus: An important cause of acute gastroenteritis in children. Lancet Child Adolesc. Health.

[44] Machiels J.D., Cremers A., van Bergen-Verkuyten M., Paardekoper-Strijbosch S., Frijns K., Wertheim H., Rahamat-Langendoen J., Melchers W. (2020). Impact of the BioFire FilmArray gastrointestinal panel on patient care and infection control. PLoS ONE.

[45] Priyanka B., Patil R.K., Dwarakanath S. (2016). A review on detection methods used for foodborne pathogens. Indian, J. Med. Res..

[46] Valledor S., Valledor I., Gil-Rodríguez M.C., Seral C., Castillo J. (2020). Comparison of several Real-Time PCR Kits versus a Culture-dependent Algorithm to Identify Enteropathogens in Stool Samples. Sci. Rep..

[47] Melendez J.H., Frankel Y.M., An A.T., Williams L., Price L.B., Wang N.Y., Lazarus G.S., Zenilman J.M. (2010). Real-time PCR assays compared to culture-based approaches for identification of aerobic bacteria in chronic wounds. Clin. Microbiol. Infect..

[48] Freeman K., Mistry H., Tsertsvadze A., Royle P., McCarthy N., Taylor-Phillips S., Manuel R., Mason J. (2017). Multiplex tests to identify gastrointestinal bacteria, viruses and parasites in people with suspected infectious gastroenteritis: A systematic review and economic analysis. Health Technol. Assess..

[49] Murugan D., Anandan S., Veereraghavan B. (2022). Real-time multiplex PCR assay reveals the increased prevalence of *Campylobacter* spp and diarrhoeagenic *Escherichia coli* in humans from Vellore, South India. J. Med. Microbiol..

[50] Pérez-Corrales C. (2019). and Leandro-Sandí, K. Diarrheagenic Escherichia coli in Costa Rican children: A 9-year retrospective study. BMC Res. Notes.

[51] Spina A., Kerr K.G., Cormican M., Barbut F., Eigentler A., Zerva L., Tassios P., Popescu G.A., Rafila A., Eerola E. (2015). Spectrum of enteropathogens detected by the FilmArray GI Panel in a multicentre study of community-acquired gastroenteritis. Clin. Microbiol. Infect..

[52] Andersson M., Kabayiza J.C., Elfving K., Nilsson S., Msellem M.I., Mårtensson A., Björkman A., Bergström T., Lindh M. (2018). Coinfection with Enteric Pathogens in East African Children with Acute Gastroenteritis-Associations and Interpretations. Am. J. Trop. Med. Hyg..

[53] Ardissino G., Possenti I., Salardi S., Tel F., Colombo E., Testa S., Daprai L., Picicco D., Colombo R.M., Torresani E. (2014). Co-infection in children with bloody diarrhea caused by Shiga toxin–producing Escherichia coli: Data of the North Italian HUS Network. J. Pediatric Gastroenterol. Nutr..

[54] Klein E., Stapp J., Clausen C., Boster D., Wells J., Qin X., Swerdlow D., Tarr P.J. (2002). Shiga toxin-producing *Escherichia coli* in children with diarrhea: A prospective point-of-care study. Pediatrics.

[55] Lindsay B., Ramamurthy T., Gupta S.S., Takeda Y., Rajendran K., Nair G.B., Stine O.C. (2011). Diarrheagenic pathogens in polymicrobial infections. Emerg. Infect. Dis..

[56] Adewakun A.A., Percival T.M., Barclay S.R., Amaechi B.T. (2005). Caries status of children in eastern Trinidad, West Indies. Oral. Health Prev. Dent..

[57] Esnard-Flavius T., Aziz Z. (2011). Microcredit, Microenterprises and Social Welfare of the Rural Poor in North-Eastern Trinidad: An Evaluation Of “Hope”. Asian Acad. Manag. J..

[58] Trinidad and Tobago Meteorological Services (TTMS) (2018). Devastating Floods of October 2018: The Mother of All Floods. http://www.cmo.org.tt/Presentations/2018/Trinidad_and_Tobago-2018.pdf.

[59] Kabore H., Levallois P., Michel P., Payment P., Dery P., Gingras S. (2010). Association between potential zoonotic enteric infections in children and environmental risk factors in Quebec, 1999–2006. Zoonoses Public Health.

[60] Levesque S., Fournier E., Carrier N., Frost E., Arbeit R.D., Michaud S. (2013). Campylobacteriosis in urban versus rural areas: A case-case study integrated with molecular typing to validate risk factors and to attribute sources of infection. PLoS ONE.

[61] Popovic-Uroic T. (1989). *Campylobacter jejuni* and *Campylobacter coli* diarrhoea in rural and urban populations in Yugoslavia. Epidemiol. Infect..

[62] Rodríguez-Lázaro D., Cook N., Ruggeri F.M. (2012). Virus hazards from food, water, and other contaminated environments. FEMS Microbiol. Rev..

[63] Su C.P., de Perio M.A., Fagan K., Smith M.L., Salehi E., Levine S., Gruszynski K., Luckhaupt S.E. (2017). Luckhaupt. Occupational distribution of Campylobacteriosis and Salmonellosis cases—Maryland, Ohio, and Virginia, 2014. MMWR-Morb. Mortal. Wkly. Rep..

[64] Croxen M.A., Law R.J., Scholz R., Keeney K.M., Wlodarska M., Finlay B.B. (2013). Recent advances in understanding enteric pathogenic *Escherichia coli*. Clin. Microbiol. Rev..

[65] Dedeić-Ljubović A., Hukić M., Bekić D., Zvizdić A. (2009). Frequency and distribution of diarrhoeagenic Escherichia coli strains isolated from pediatric patients with diarrhoea in Bosnia and Herzegovina. Bosn. J. Basic Med. Sci..

[66] Huiwen Deborah Chen and Gad Frankel (2005). Enteropathogenic Escherichia coli: Unravelling pathogenesis. FEMS Microbiol. Rev..

[67] Panlozzi L.J., Johnson K.E., Kamahele L.M., Clausen C.R., Riley L.W., Helgerson S.D. (1989). Diarrhoea associated with adherent enteropathogenic Escherichia coli in an infant and toddler centre, Seattle, Washington. Pediatrics.

[68] Bokhari H., Shah M.A., Asad S., Akhtar S., Akram M., Wren B.W. (2013). *Escherichia coli* Pathotypes in Pakistan from Consecutive Floods in 2010 and 2011. Am. Soc. Trop. Med. Hyg..

[69] Ochoa T.J., Contreras C.A. (2011). Enteropathogenic Escherichia coli infection in children. Curr. Opin. Infect. Dis..

[70] Shetty V.A., Kumar S.H., Shetty A.K., Karunasagar I., Karunasagar I. (2012). Prevalence and characterization of diarrheagenic Escherichia coli isolated from adults and children in Mangalore, India. J. Lab. Physicians.

[71] Trabulsi L.R., Keller R., Tardelli Gomes T.A. (2002). Typical and atypical enteropathogenic Escherichia coli. Emerg. Infect. Dis..

